# Green Crab (*Carcinus maenas*) Foraging Efficiency Reduced by Fast Flows

**DOI:** 10.1371/journal.pone.0021025

**Published:** 2011-06-07

**Authors:** Elizabeth M. Robinson, Delbert L. Smee, Geoffrey C. Trussell

**Affiliations:** 1 Department of Life Sciences, Texas A&M University – Corpus Christi, Corpus Christi, Texas, United States of America; 2 Center of Marine Science, Northeastern University, Nahant, Massachusetts, United States of America; University of Hull, United Kingdom

## Abstract

Predators can strongly influence prey populations and the structure and function of ecosystems, but these effects can be modified by environmental stress. For example, fluid velocity and turbulence can alter the impact of predators by limiting their environmental range and altering their foraging ability. We investigated how hydrodynamics affected the foraging behavior of the green crab (*Carcinus maenas*), which is invading marine habitats throughout the world. High flow velocities are known to reduce green crab predation rates and our study sought to identify the mechanisms by which flow affects green crabs. We performed a series of experiments with green crabs to determine: 1) if their ability to find prey was altered by flow in the field, 2) how flow velocity influenced their foraging efficiency, and 3) how flow velocity affected their handling time of prey. In a field study, we caught significantly fewer crabs in baited traps at sites with fast versus slow flows even though crabs were more abundant in high flow areas. This finding suggests that higher velocity flows impair the ability of green crabs to locate prey. In laboratory flume assays, green crabs foraged less efficiently when flow velocity was increased. Moreover, green crabs required significantly more time to consume prey in high velocity flows. Our data indicate that flow can impose significant chemosensory and physical constraints on green crabs. Hence, hydrodynamics may strongly influence the role that green crabs and other predators play in rocky intertidal communities.

## Introduction

Predators often have large effects on the structure and function of aquatic and terrestrial communities [1], [2] by consuming prey [2], [3] and by initiating trophic cascades that affect the abundance of resources within a food chain [4]–[6]. Although these effects are well appreciated, environmental forces can modify predator foraging activities and have large effects on predation rates and community dynamics. For example, mobile predators are often absent on wave-swept shores as hydrodynamic stress associated with waves prevents them from foraging effectively and poses risk of injury and death [7], [8]. In these situations, stress may act as the primary agent of community regulation and render biotic effects, such as predation, unimportant.

Yet, stress may also influence communities by modifying predatory interactions at levels that interfere with the behaviors of predators and/or prey but are otherwise benign [9]–[11]. For example, green crab predation rates are significantly greater in estuarine habitats with slow flow velocities, and decline significantly as velocity increases [12], [13]. Green crab densities are greater in high flow sites than low flow sites [13], indicating that these flow velocities do not prevent crabs from inhabiting the area but do reduce green crab foraging. Likewise, in freshwater systems, slight increases in turbidity can alter the outcomes of predatory interactions and influence indirect predator effects and trophic relationships [14]. Like turbidity and flow, substrate type and gas concentrations can reduce the foraging success of predators [15]–[17], providing a potential niche for stress-tolerant organisms [1], [7], [18].

Hydrodynamic stress can influence predation rates by limiting predator mobility [19], foraging efficiency [20], chemosensory functioning [21], or in extreme cases, prevent predators from inhabiting an area [22]. In marine systems, organisms often depend upon chemical signals for foraging and predator avoidance, but the delivery and detection of chemical odor plumes is strongly influenced by hydrodynamic properties such as flow velocity and turbulence [23], [24]. Fast and/or turbulent flows increase mixing of chemical signals, homogenize odor plumes, increase plume width, and decrease the range of concentration of odor filaments within the plume [9], [23], [25]. By altering chemical signal structure, turbulent flows can strongly affect the chemoreceptive abilities of organisms [21], [26].

Predicting the effects of different hydrodynamic regimes on predator-prey interactions is not always straightforward because animals may use a variety of strategies for tracking chemical odor plumes, leading to varying degrees of foraging success in different flow conditions [26]. For example, terrestrial organisms like moths use a combination of visual and chemical signals to follow airborne chemical plumes [26], [27]. They move up wind in the direction of the plume's source and use visual cues to insure they are making progress moving into the wind [27]. Aquatic crustaceans are not known to utilize visual cues to assist in navigating through waterborne chemical plumes and instead depend upon combinations of rheotaxis and chemotaxis to locate sources of these chemical cues [11], [26], [28]. Lobsters (*Homarus americanus*) and crayfish (*Orconectes rusticus*) rely on information contained within odor plumes, such as the frequency between concentrated odor filaments, to mediate upstream movement and are not adversely affected by increased turbulence [28]–[30]. In contrast, blue crabs (*Callinectes sapidus*) use ‘odor-guided rheotaxis’ while foraging, where crabs detect cue and move up stream [21], [31]. Previous work with blue crabs has shown they are unable to successfully orient to chemical sources in no-flow conditions and are less efficient and successful when flow velocity or turbulence increases [9]–[11], [19]. Both crabs and lobsters utilize spatial sampling of odor plumes to remain within the plume as they move upstream toward attractive chemical cues [11], [26], [28], [32]. In contrast to blue crabs, knobbed whelks (*Busycon carica*) are better able to find prey and become more efficient predators in more turbulent flows despite their limited spatial sampling ability [10], [33], [34]. Previous authors [10], [21], [33], [34] hypothesized that slower-moving organisms, such as knobbed whelks and other gastropods, may utilize a temporal integration strategy for chemosensory foraging to compensate for their poor spatial sampling capability, which helps them forage in turbulent conditions. In addition, flow may also affect the ability of prey to react to predators and increase predation rates and alter the spatial extent of nonconsumptive predator effects [35]–[37].

Besides affecting chemosensory functioning, flow may impose physical limitations on organisms such as drag. Drag is a force that opposes relative motion of an organism through a fluid and is dependent upon velocity; with an increase in flow velocity, organisms can experience an increase in drag. Organisms have developed flexible, streamlined bodies to reduce drag imposed by moving fluids [38] or may change their behavior to lessen drag. For example, as flow velocity increases blue crabs adopt a drag-minimizing posture (i.e. move sideways), which reduces locomotory costs but places their sensory organisms in a position that hinders chemoreception [19].

The goal of this research was to determine how hydrodynamics influence the foraging ability of a common intertidal predator by affecting prey-finding ability and imposing physical limitations on prey handling. Using European green crabs (*Carcinus maenas*) as a model organism, we investigated how flow velocity modified foraging behavior. Results from field and laboratory experiments suggest that fast flows reduce the prey-finding ability of green crabs and decrease green crab ability to handle and consume prey.

## Methods

### Description of Model Organism and Study System

The green crab is an invasive species that competes with other native and invasive crab species for a variety of prey species, including mussels, snails, clams, and scallops [39]–[43]. Its native range extends from Northern Africa to Norway and it has invaded the coasts of North America, South America, Australia, and South Africa [43]. Where large populations of green crabs exist, there are often reductions in biodiversity [39]–[41]. Green crabs were selected because they are abundant predators in rocky intertidal communities and have significant effects on community structure [6], [44]–[47].

Green crab predation declines in faster flows, altering succession patterns and community assemblages in rocky intertidal systems [12], [13]. Green crab densities along the Damariscotta River, Maine, USA are greater in high flow environments than in low flow environments, but predation on mussels and snails is lower in high flow areas despite the higher abundance of crabs. High flow sites are often dominated by mussel beds and have little open space for colonization while high predation on mussels by green crabs at low flow sites creates a community dominated by seaweeds that has considerable open space [12], [13].

### Field Study

**Figure 1:**
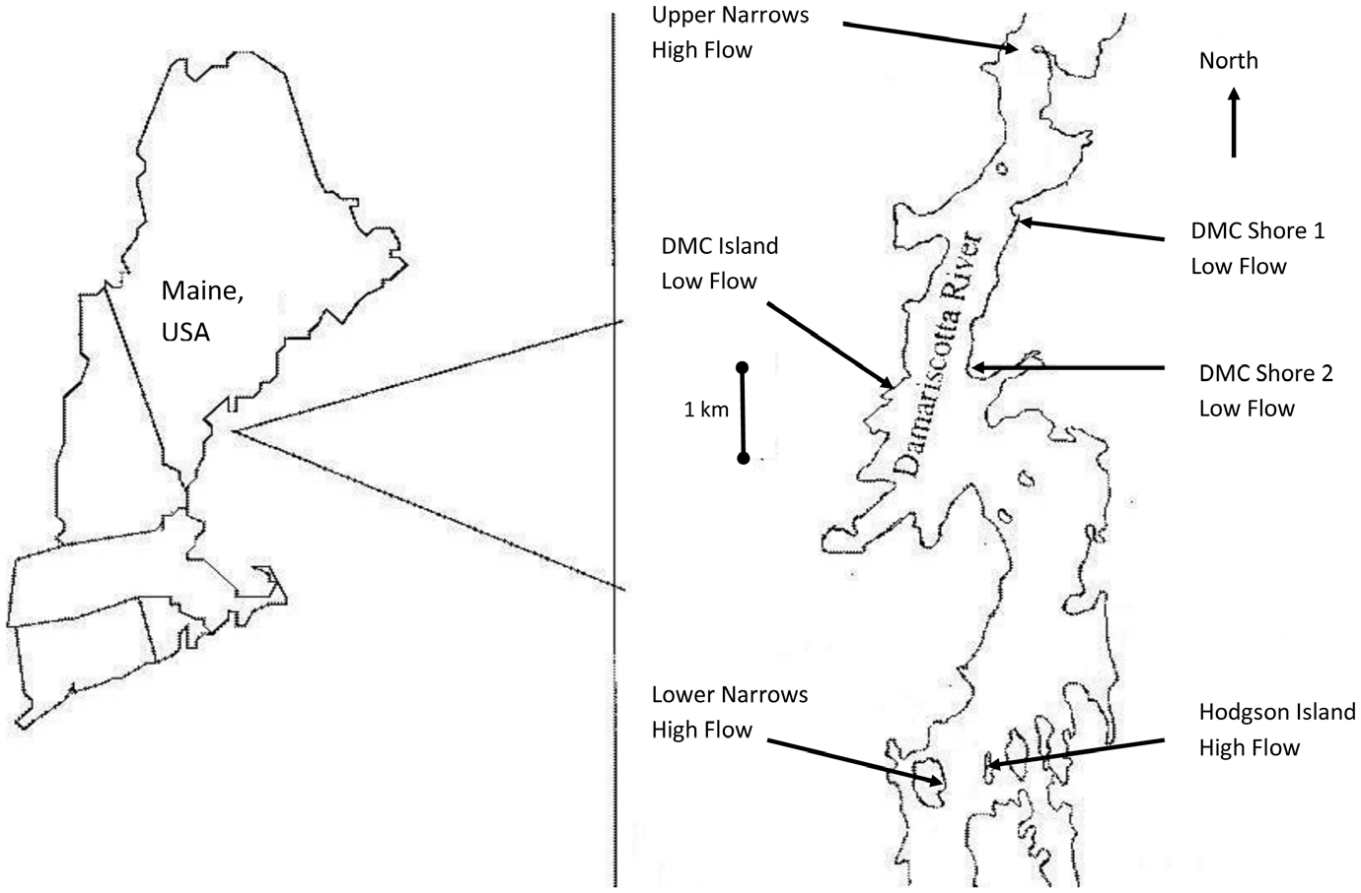
The location of field sites along the Damariscotta River, Maine, USA. Field sites were used in the trapping study to determine if foraging is reduced in high flow sites vs. low flow sites. Map modified from Leonard et al. (1998).

We examined the influence of flow velocity and turbulence on the foraging ability of green crabs in the field at sites in the Damariscotta River, Maine, USA. To determine if green crab ability to detect and find chemical cues is altered by flow in the field, we conducted an experiment to see if crabs were more likely to enter baited traps in high vs. low flow areas. Crab traps (volume = 0.5 m^3^) were constructed with vexar mesh (1.0 cm^2^ openings) and were secured ∼1.0 m above mean lower low water (MLLW) in the rocky intertidal zone using metal anchors. Green crabs migrate and forage in intertidal areas during flood tide, and may travel over 150 m during a single flood tide [48] before retreating with the ebb tide to reduce predation risk and desiccation stress. We sampled three low flow sites and three high flow sites in the Damariscotta River (Figure 1). Six pots were placed in the field during low tide in groups of three so that three traps were in a high flow site and three in a low flow site each day. Traps were baited with six crushed mussels and placed ∼50 m apart at each site and were recovered after 24 hours to count and remove crabs. The traps were moved to different sites every 24-h, but we always placed 3 traps in high flow and 3 in low flow sites to avoid temporal bias in our results. We measured the ability of crabs to locate attractive prey chemical cues in the field by counting the total number of crabs in each pot. Data between sites were compared using a t-test [49].

### Field Hydrodynamic Measurements

We measured flow conditions in each field site using Vector model acoustic Doppler velocimeters (ADVs, NortekUSA™) and vendor supplied ExploreV™ software. ADVs were deployed in each field site during low tide ∼0.50 m above the mean lower low water line and measured flow velocity 0.50 m above the substrate. Flow velocity was sampled at a frequency of 16 Hz in 4-minute bursts every 15 minutes for 24 hours. All data collected when the ADVs were out of water was discarded. Because we used six field sites but only possess 4 ADVs, we rotated the instruments between sites during a 4 day period so that each site was measured 3×. Two low flow and two high flow sites were measured each day.

ADVs measure 3 dimensional flows, and we calculated the net flow velocity (U) using the formula U = √(u^2^+v^2^+w^2^) where u, v, and w are the velocity components in the x, y, and z dimensions respectively. We determined the net flow velocity for each 4 minute measurement period and then averaged all of the measurement periods to determine the mean flow velocity for each site. Turbulence was calculated using the root mean square (RMS) of the velocity time series. As with flow velocity, we combined RMS in the x, y, and z dimensions for each 4 minute measurement period using the formula RMS = √(RMS_u_^2^+RMS_v_^2^+RMS_w_^2^) where these values represent the RMS levels in the x, y, and z dimensions respectively. We then averaged these RMS calculations from all measurement periods to determine the turbulence levels in each field site. We also reported the min and max flow velocity records from each field site.

### Animal Capture

Green crabs were collected from the rocky intertidal zone of the Damariscotta River by hand and with baited crab pots. Dogwhelks (*Nucella lapillus*) and blue mussels (*Mytilus edulis*) were collected by hand on exposed intertidal shoreline at low tide. These organisms were shipped overnight to Texas A&M University – Corpus Christi, Corpus Christi, Texas (TAMU-CC) in refrigerated containers. These organisms were kept in insulated aquaria with recirculated, filtered seawater at ∼13°C and a salinity of 35. Green crabs were fed blue mussels once per week and not fed 48 h before use in behavioral assays. All animals were used in a single assay and then humanely destroyed by freezing and disposed of in a land-based facility in compliance with IACUC protocol.

### Hydrodynamic Environment

To determine how flow velocity affected the foraging behavior of green crabs, we performed behavioral assays in a recirculating flume at TAMU-CC. The flume was 4.25-m long×0.75-m wide×20.0 cm deep and can reliably produce flows from 1–20 cm s^−1^. Flume water was filtered daily using a 50-mm biological filter and was maintained at a temperature of 13°C and salinity of 35. Behavioral assays were performed under artificial light conditions. We performed preliminary assays in a small flume at the Darling Marine Center (DMC) in Walpole, ME. This flume was useful to develop our behavioral assays, but the hydrodynamic environment is quite different than the flume at TAMU-CC, and thus trials performed in both flumes were not statistically compared. We did note however that crabs assayed at TAMU-CC displayed similar behaviors to those assayed at the DMC, alleviating our concerns that shipment to Texas and housing in non-flowing sea water might affect crab behaviors.

### Laboratory Hydrodynamic Measurements

Flow conditions in the TAMU-CC flume were measured using a Vectrino model Acoustic Doppler Velocimeter (ADV, NortekUSA™). Free-stream velocity (*U*), shear velocity (U*), and the root mean square of flow velocity (RMS) are commonly used to quantify flow environments [11], [44]. The net flow velocity (U) was calculated using the formula U = √(u^2^+v^2^+w^2^) as previously described, and turbulence was calculated using the root mean square (RMS) of the velocity time series. As with flow velocity, RMS was combined in the x, y, and z dimensions using the formula RMS = √(RMS_u_^2^+RMS_v_^2^+RMS_w_^2^).

Free-stream velocity in the flume was measured 11 cm above the substrate. Turbulence (RMS) was quantified by measuring flow at 10 Hz 4.0 cm from the substrate. This measurement height was selected to quantify turbulence because it is within a height typically sampled by green crab antennules. Shear velocity is a measure of how much momentum is transferred into the boundary layer and is indicative of levels of near-substrate turbulence [37], [50], [51]. Shear velocity was calculated by measuring flow with the ADV at 12 heights within the log layer region of the boundary layer (i.e., the first 30%, or 6 cm extending from the substrate). Flow velocity was measured at each height for 2 min at a sampling rate of 10 Hz. Shear velocity was calculated by regression fit using the Karman-Prandtl equation (“law of the wall”) from the ADV data collected at different heights [51]. All regressions used to calculate shear velocities had r^2^>0.95.

### Behavioral Assay

Green crabs were placed in a cage (0.3 m×0.3 m×0.46 m) located in the flume 1.0 m downstream from a single crushed mussel (*Mytilus edulis*) (12.5–17.5 g). Although this cue is likely stronger than a cue a green crab would receive in the field, a strong, but consistent cue was required to ensure that the crabs would respond in all flow conditions. In preliminary studies, crabs would not track consistently to live, uninjured prey. Moreover, we used crushed mussels in our field experiment and wanted to continue to use a similar cue for flume behavioral assays. Since the purpose of these assays was to determine how flow affects foraging behavior and not to determine the ecological concentrations of cues, a more concentrated cue was used to elicit crab foraging responses. Only male crabs were used in experiments to prevent differences in behavior due to sex [52], [53]. The crabs assayed had a carapace width of 60–75 mm.

Behavioral assays began by placing an individual crab 1.0 m downstream from the crushed mussel for a 10-min acclimation period. After 10 min, they were released and allowed to travel upstream towards the mussel. The behavioral assay was terminated when the crab found the mussel (successful), touched the side of the flume, moved upstream past the mussel, or remained in the flume for 5 min without finding the mussel. Only crabs successfully locating the mussel were used in analysis.

Behavioral assays were performed at two flow velocities: 15 cm s^−1^ and 19 cm s^−1^. These flow velocities were used for several reasons. First, these flows are representative of natural conditions green crabs experience in the field (Table 1). Second, in low flow sites, our ADV measurements indicated that flow velocity was below 15 cm s^−1^ in more than 80% of our measurements. In contrast, velocity was below 15 cm s^−1^ in less than 40% of our measurements in high flow sites. Finally, in preliminary behavioral assays, green crabs displayed similar foraging behaviors in flows of 3 cm s^−1^ and 15 cm s^−1^, suggesting that their movement towards the stimulus source we used would be similar in all flows lower than 15 cm s^−1^. The higher flow velocity of 19 cm s^−1^ was selected because it is the upper velocity limit of our flume, and we wanted to provide a large contrast in flow conditions to assess differences in prey-finding behaviors.

**Table 1:** Hydrodynamic conditions measured in 6 field sites in the Damariscotta River, ME, USA.

Site	Mean Flow cm s^−1^	Min Flow cm s^−1^	Max Flow cm s^−1^	Mean RMS
Upper Narrows (High Flow)	57.7	0.4	73.0	13.4
Hogson Island (High Flow)	23.2	1.4	69.4	17.0
Lower Narrows (High Flow)	34.8	0.7	119.1	12.6
DMC Shore 1 (Low Flow)	10.5	1.2	23.2	9.5
DMC Shore 2 (Low Flow)	5.0	0.6	48.0	9.7
DMC Island (Low Flow)	7.7	0.7	16.3	5.7

Behavior of the green crabs was recorded using a Panasonic™ PV-GS35 camera placed to capture the 1-m test area between the crab and the prey. Crabs were outfitted with two chemo-luminescent beads along the widest, horizontal axis of their carapace. Video was recorded at 25 Hz and digitized using a Vicon Motus Motion Analysis© software. Previous studies examining blue crab tracking behavior have used video data collected at 2 Hz and 5 Hz [9], [32], and interpretation of raw data may be affected by video rates. We therefore down sampled our data and measured differences in behavioral parameters when using rates of 2, 5, and 25 Hz. We did not find significant differences in the behavioral parameters reported and thus used our raw data collected at 25 Hz, which we report in this paper.

The following variables were calculated from the crab videos and the Vicon system to estimate search efficiency: the period of time it took the green crab to locate its prey (foraging time, s), the walking speed (cm s^−1^) of the green crab towards the prey, and the distance traveled (cm) by the green crab to its prey. The variables are defined as follows: foraging time was the time from when the crab was released from the cage to the touching of the prey; distance traveled was the total path distance the crab moved towards the prey; and walking speed toward the source was the change in distance from the starting location per unit of time [11], [29], [32]. Longer foraging times and slower walking speeds are indicative of difficulty in finding the source of chemical cues and suggest a lower foraging efficiency. Longer distances traveled show that green crabs are casting from side to side as they move upstream, which is less efficient than moving straight to the odor source, and suggest greater difficulty and less efficiency in following an odor plume [32].

Each specific parameter of green crab foraging behavior (walking speed, distance traveled to source, and time to find source) was analyzed separately using a t-test [49].

### Handling Time

To determine how flow affects the prey handling time of green crabs, individual crabs were placed within a vexar mesh cage (1.0 m×0.5 m×0.5 m with 1.0 cm^2^ openings) inside the TAMU-CC flume. Crabs were acclimated for 10 minutes in the cage, after which time a single dogwhelk was placed ∼1.0 cm from the crab's mouthparts. Handling time in seconds was recorded from the crab's initial contact with the dogwhelk through consumption. Consumption was deemed complete when the green crab walked away from the dogwhelk. We did not observe crabs leaving the dogwhelk until all visible soft tissue was consumed. Since dogwhelk shell thickness and size may vary geographically [54], [55], all dogwhelks used for this experiment were collected from the same geographic area and were 20–25 mm in length. Crabs were given a maximum time of 20 min to consume the dogwhelk. Ten assays were performed under free-stream flow velocities of 3 cm s^−1^, 15 cm s^−1^, and 19 cm s^−1^. Handling time was compared between flow velocities using a one-way ANOVA with flow velocity as a fixed factor [49]. Tukey-Kramer post-hoc analysis was used for pair wise comparisons between means [49].

## Results

### Field Experiment

**Figure 2:**
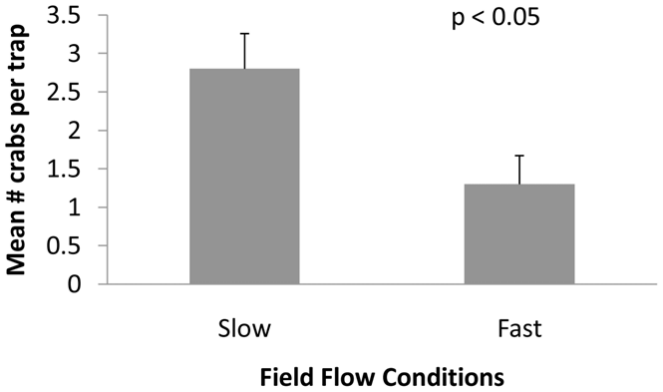
Mean number (± SE) of *C. maenas* caught per trap in field locations with low and high flow velocities. Means were compared using a t-test and were significantly different (p<0.05, n = 19).

Mean flow velocities were 3 fold greater in high flow as compared to low flow sites. RMS and max flow velocities were also greater in high flow areas (Table 1). The total number of green crabs caught in high flow sites was significantly lower than those caught in low flow sites (t = 2.44, df = 36, *p*<0.05; Figure 2), even though green crab density is known to be greater in these faster flow sites.

### Flume Assays

**Figure 3:**
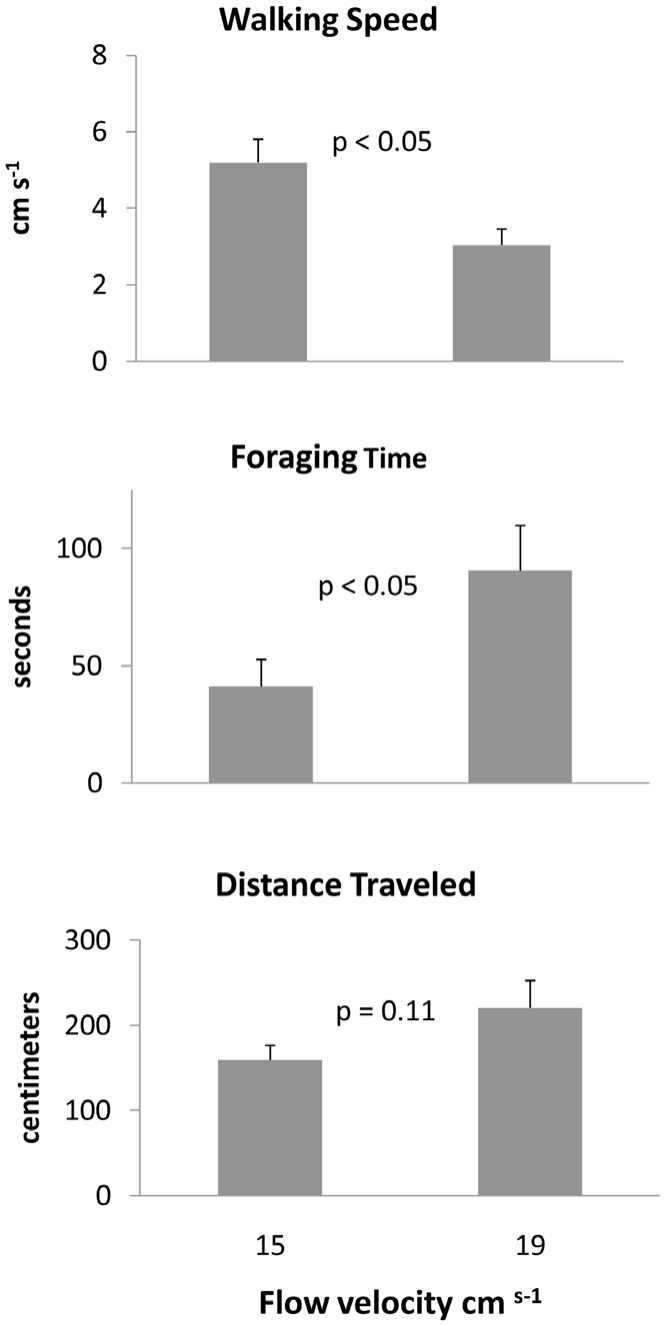
Behavioral parameters measured in flume assays of successfully foraging green crabs in two flow velocities. Graphs show mean (±SE) of: walking speed (cm s^−1^), foraging time (seconds), and total distance traveled (centimeters). T-tests were performed for each parameter, p values are shown on each graph with significance a α = 0.05, and n = 10.

**Figure 4:**
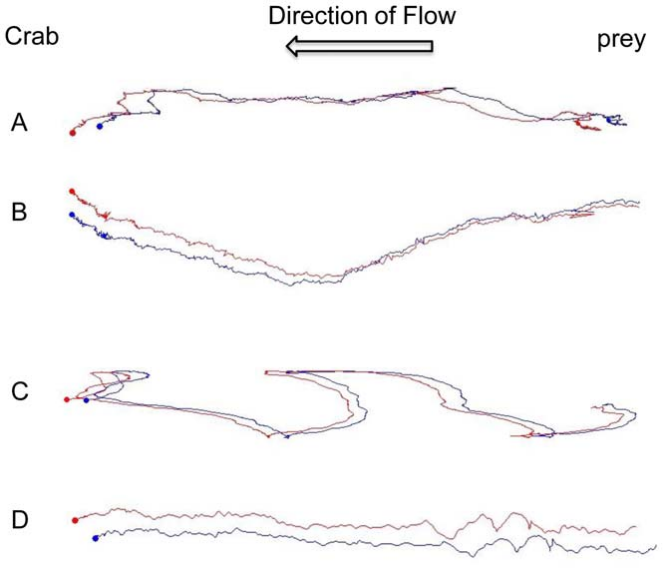
Example path trajectories of successful, foraging green crabs in the flume at *U* = (A) 3 cm s^−1^ tile, (B) 15 cm s^−1^ tile, and (C) 19 cm s^−1^ tile. An example of an unsuccessful green crab is presented as trajectory (D). The two lines for each trajectory represent the two tracking markers located on the carapace of the green crab. 3 cm s^−1^ trials were performed in the DMC flume.

As free-stream velocity increased from 15 cm s^−1^ to 19 cm s^−1^, RMS increased from 1.79 cm s^−1^ to 1.89 cm s^−1^ and shear velocity increased from 2.79 cm s^−1^ to 3.66 cm s^−1^ (Table 2). Flow velocity significantly affected the foraging time (t = 2.66, df = 18, *p*<0.05; Figure 3) and walking speed (t = 2.20, df = 18, *p*<0.05; Figure 3) of green crabs when successfully locating prey. Although not statistically significant, green crabs traveled ∼220 cm while foraging in the 19 cm s^−1^ flow as compared to ∼160 cm in the 15 cm s^−1^ flow (t = 1.67, df = 18, *p* = 0.12; Figures 3, 4). These results suggest that foraging efficiency is strongly affected by increased flow velocity.

**Table 2:** Hydrodynamic conditions measured in the Texas A&M University – Corpus Christi (TAMU-CC) flume.

Free stream Velocity cm s^−1^	RMS	U* (shear velocity) cm s^−1^
15	1.79	2.79
19	1.89	3.66

### Handling time

**Figure 5:**
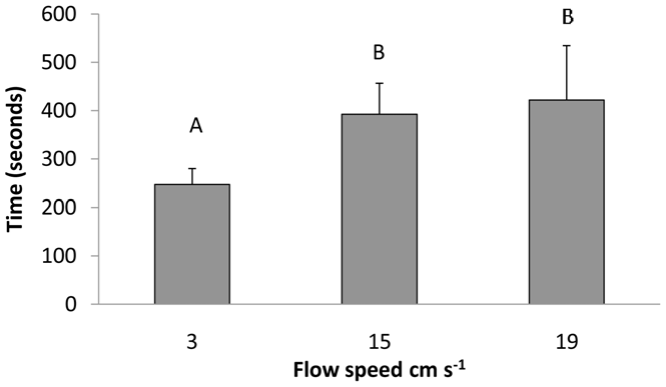
Mean handling time (±SE) of green crabs (n = 10) at flow velocities *U* = 3 cm s^−1^, 15 cm s^−1^, and 19 cm s^−1^. ANOVA indicated significant differences (p<0.05, n = 10). Letters denote significant differences passed upon a Tukey-Kramer post hoc test.

Flow velocity increased the prey handling time of green crabs. Handling time significantly increased in faster flows as green crabs took significantly longer to consume dogwhelks in *U* = 15 cm s^−1^ and 19 cm s^−1^ as compared to *U* = 3 cm s^−1^ (F_2,27_ = 5.492, *p*≤.01, Fig. 5). Thus, flow can impose physical limitations on green crabs after they have found a potential meal.

## Discussion

Flow velocity and turbulence strongly affect the advection of odor molecules in air and water environments and the ability of organisms to detect chemical cues [9], [20], [21], [26], [28], [30]. Fluid forces can also impose physical forces on animals that interfere with foraging activities (e.g., lift and drag, [19], [51]). We found that increased flow velocity hindered the ability of green crabs to locate potential meals and made it more difficult for them to consume captured prey. In crabs, foraging behaviors are generally mediated by the sensory neurons whose receptors are located in the legs and antennae [42], [56]. While green crabs have eyes designed to detect movement by variations in shadows and vibrations, they are not efficient organs for use in the foraging of prey, and instead green crabs rely heavily on their chemosensory organs [42]. Thus, green crabs are not likely to be able to overcome sensory decrements caused by flow by relying on visual signals.

In the field, green crabs experienced reduced foraging success in high flow environments (Table 1, Figure 2). Despite the higher numbers of green crabs at fast flow sites [13], we caught fewer green crabs at these locations in baited traps. Our design is limited in that we cannot determine if we caught fewer green crabs in faster flows because they had greater difficulty detecting attractive odor cues in these conditions or if they simply refused to move toward foraging cues in fast flow. Thus, differences in crab numbers caught between sites may either reflect difficulty detecting odor plumes, less frequent search attempts, or some combination thereof. Regardless, these data along with previous work in this system [12], [13] reveal that hydrodynamics can strongly influence rates of prey mortality in this system.

Increasing water velocity in the flume behavioral assays from 15 cm s^−1^ to 19 cm s^−1^ caused RMS to increase from 1.79 to 1.89 and shear velocity (U*) to increase from 2.79 to 3.66 cm s^−1^. As turbulence increases, odor plumes are mixed and the plume becomes wider and homogenized so that the chemical concentration within the plume are more uniform [9], [23]. Increased turbulent mixing may either enhance or diminish the effectiveness of aquatic chemosensory foragers depending upon the mechanisms used by organisms to follow chemical plumes [21], [26], [28], [29], [34]. For example, blue crabs show decreased search efficiency and success as turbulence increases, while other organisms including lobsters, crayfish, and knobbed whelks can successfully forage and are more efficient in more turbulent flows [10], [28], [29], [34].

In this study, successfully foraging green crabs decreased their walking speed and increased their foraging time and distance traveled to the cue source when free-stream velocity increased (Table 2, Figure 3). These changes reveal greater difficultly and less efficiency in finding sources of attractive chemical cues. Turbulent mixing causes blue crabs to move in a trajectory resembling a zigzag pattern (i.e., casting) to cover large, spatial distances in search of the chemical cues [9], [11], [32], [56]. When blue crabs reach the edge of an odor plume and the concentration is low or non-existent, they either cease movement and wait for a defined pocket of odor or immediately turn back into the odor plume [9], [32]. In contrast, lobsters and crayfish are able to utilize chemical information within odor plumes, such as the frequency between concentrated odor filaments, to mediate upstream movement and are not adversely affected by increased turbulence [28]–[30]. These organisms typically do not display casting behavior observed in crabs and moths [28]–[30]. Crayfish move in similar trajectories and travel similar distances, and may actually walk faster in more turbulent flows [29], [30]. Previous work suggests that lobsters possess a long term chemo-navigation strategy mediated by chemoreceptors on their antennules and switch to a localized search mediated by chemosensors on the legs when nearing an attractive odor source, a mechanism not observed in crabs [28].

Although we did not measure odor plume dynamics in this study, we saw similar turning behavior in green crabs (Figure 4) as have been noted by other authors using blue crabs [9], [32]. Like blue crabs, green crabs walked more slowly in faster flows, covered greater distances and took longer to reach the odor source, which is also similar to blue crabs and unlike lobsters and crayfish [9], [26], [28]–[30], [32]. We predict that green crabs have similar mechanisms for olfactory navigation as blue crabs, but additional studies beyond the scope of the present work are needed to more precisely examine green crab olfactory foraging. Our results show that increased flow velocity reduced green crabs' foraging efficiency, but our data is not sufficient to determine if reductions in foraging result from increased turbulent mixing of the odor plume in faster flows, faster advection of odor molecules, or other physical limitations. Future work is needed to separate the effects of turbulence from flow velocity on green crab foraging efficiency using as has been elegantly done for blue crabs [9], [32] and crayfish [29].

Although green crabs were hindered by faster flows, they were able to successfully track to prey when velocity was 19 cm s^−1^ and shear was >3.5 cm s^−1^. Blue crabs show significantly reduced foraging performance when shear velocities reach 0.05 cm s^−1^, two orders of magnitude below that tested in this study (Weissburg and Zimmer-Faust 1994, Jackson et al. 2007). Green crabs inhabit environments with flows much faster than those encountered by blue crabs. The slowest flow sites in Maine had similar velocities to the fastest flow sites used in a recent field study with blue crabs in Georgia [12], [13], [36]. Thus, the range of flow conditions that green crabs can successfully forage in may be much broader than that of blue crabs. Chemical navigation by lobsters and crayfish has not been investigated in the fast flow velocities used in this study.

While green crabs were able to forage in faster flows than blue crabs, and in faster flows than have been tested for lobsters and crayfish, it is important to note the differences in chemical cue concentrations used in this study compared to previous studies. We used chemical cues released from a crushed mussel, whereas previous authors [11] used live, actively pumping clams (*Mercenaria merceneria*), effluent made by soaking prey tissue in a known volume of seawater [9], [28], or fish extract placed in gelatin [29]. Future studies comparing foraging across several cue concentrations and flow conditions would be useful to more thoroughly compare green crab and blue crab foraging abilities. Both crabs are known to co-occur in shallow water habitats of bays and estuaries along Maryland, New Jersey, Rhode Island, and Massachusetts causing overlap in habitat utilization and diet [39], [40], [57]. Additional studies with lobsters and crayfish in flows with the velocities used here would also be useful in evaluating their upper limits for successful and efficient navigation.

Unlike the field experiments, no change in foraging success rates occurred across flow treatments in flume assays. This result may have emerged because the flume is incapable of producing flow velocities above 20 cm s^−1^ that are common in high flow areas of the Damariscotta River [13] or because the concentration of chemical cues used in the flume was high enough that hydrodynamic mixing did not reduce odor concentrations below detectable thresholds. We used crushed mussels as the attractive cue source in flume assays to elicit crab foraging in all flow treatments because preliminary studies with live prey under fast flow conditions did not elicit successful green crab foraging behavior. Despite the cue's high concentration, we found that faster and more turbulent flows reduced foraging efficiency (Figure 3). We also used crushed mussels as the cue source in the field study, suggesting that successfully detection of crushed mussels by green crabs can be influenced by flow.

Since we needed this strong cue to coax crabs upstream in behavioral assays and green crabs are more abundant in field sites with fast flows, we hypothesized that green crabs may not detect and track toward single prey items in the field. Instead, they may be tracking to a mussel bed community that produces a large chemical signature, which the crabs may be able to detect in higher velocity flows. By tracking toward a mussel bed where prey are likely to be abundant, green crabs would have a high probability of finding a suitable meal. Green crabs have been shown to migrate to foraging habitats during high tide [55], and a stronger community cue may be present in the environment to initiate this movement. Predators tend to congregate near areas of dense prey (by reproduction or by migration) because of high sources of nourishment [58], and mussel recruitment and mussel density is greater in high flow than low flow areas [12], [13].

In New England rocky intertidal systems, flow imposes sensory and physical limitations on green crabs, and by altering predation rates, can have large effects on the structure of communities. Turbulence and velocity are both important in the restriction of predators by limiting sensory detection and increasing drag. Overall, for predators that are traveling longer distances to feed (>1.0 m), prey detection is likely hindered significantly by turbulent mixing that makes cues more difficult to detect. For localized foragers that live in high flow environments with abundant prey, prey detection and location may prove less challenging and drag may have a larger effect on predation by reducing prey-handling efficiency. Clearly additional work is needed to assess the effects of flow on predatory interactions at varying predator and prey densities.

Classic models of community organization [7] emphasize the regulation of community structure through biological interactions (species interactions, larval production) and/or environmental stress (desiccation and wave forces). Our study indicates that relatively benign environmental variation can dictate foraging efficiency by influencing the ability of predators to find and handle prey. When changes in environmental stress are relatively small (i.e., change in flow velocity from 15 cm s^−1^ to 19 cm s^−1^), green crab foraging efficiency was significantly reduced as was handling time when flow velocity increased from 3 cm s^−1^ to 15 cm s^−1^. Recent studies suggest that flow may also affect prey ability to avoid predators [33], [35]–[37], and more work is needed in this system to understand how both predators and prey are affected by environmental conditions like flow.

Predator-prey interactions have been studied exhaustively over the past half century, but much of this work has yet to address how abiotic processes that are common to marine benthic systems, such as hydrodynamics, influence the outcomes of predatory interactions and ultimately top-down forces and community structure. While this study focused on flow, other environmental features such as turbidity may also influence the outcomes of predatory interactions, and by influencing predation rates, exert considerable influence over the structure and function of communities. Future studies addressing how environmental context influences the outcomes of predatory interactions will likely provide key insights as to the combined influence of biotic and abiotic factors in structuring communities.
